# The Effect of Gestational Age on Angiogenic Gene Expression in the Rat Placenta

**DOI:** 10.1371/journal.pone.0083762

**Published:** 2013-12-31

**Authors:** Kanchan Vaswani, Melissa Wen-Ching Hum, Hsiu-Wen Chan, Jennifer Ryan, Ryan J. Wood-Bradley, Marloes Dekker Nitert, Murray D. Mitchell, James A. Armitage, Gregory E. Rice

**Affiliations:** 1 University of Queensland Centre for Clinical Research, Royal Brisbane and Women’s Hospital Campus, Herston, Queensland, Australia; 2 School of Medicine (Optometry), Deakin University, Waurn Ponds, Victoria, Australia; 3 Department of Anatomy & Developmental Biology, Monash University, Clayton, Victoria, Australia; Cincinnati Children’s Hospital Medical Center, United States of America

## Abstract

The placenta plays a central role in determining the outcome of pregnancy. It undergoes changes during gestation as the fetus develops and as demands for energy substrate transfer and gas exchange increase. The molecular mechanisms that coordinate these changes have yet to be fully elucidated. The study performed a large scale screen of the transcriptome of the rat placenta throughout mid-late gestation (E14.25–E20) with emphasis on characterizing gestational age associated changes in the expression of genes invoved in angiogenic pathways. Sprague Dawley dams were sacrificed at E14.25, E15.25, E17.25 and E20 (n = 6 per group) and RNA was isolated from one placenta per dam. Changes in placental gene expression were identifed using Illumina Rat Ref-12 Expression BeadChip Microarrays. Differentially expressed genes (>2-fold change, <1% false discovery rate, FDR) were functionally categorised by gene ontology pathway analysis. A subset of differentially expressed genes identified by microarrays were confirmed using Real-Time qPCR. The expression of thirty one genes involved in the angiogenic pathway was shown to change over time, using microarray analysis (22 genes displayed increased and 9 gene decreased expression). Five genes (4 up regulated: *Cd36, Mmp14, Rhob and Angpt4* and 1 down regulated: *Foxm1*) involved in angiogenesis and blood vessel morphogenesis were subjected to further validation. qPCR confirmed late gestational increased expression of *Cd36, Mmp14, Rhob* and *Angpt4* and a decrease in expression of *Foxm1* before labour onset (P<0.0001). The observed acute, pre-labour changes in the expression of the 31 genes during gestation warrant further investigation to elucidate their role in pregnancy.

## Introduction

A successful outcome to pregnancy depends on establishing an effective materno-fetal exchange interface. Two processes critical for materno-fetal exchange are adequate perfusion of the placenta by maternal blood, and formation of the placental villous tree (the exchange surface of the placenta) and its vascular network. Failure to achieve either of these processes is associated with adverse pregnancy outcome, including: miscarriage; intrauterine growth restriction; preeclampsia and preterm delivery. Furthermore, epidemiological studies identify a strong association between compromised placental structure and function, impaired fetal growth and the development of adult-onset diseases [1]–[3].

Development of the vascular network within placental villi during first trimester is a critical process that involves both vasculogenesis and angiogenesis. In humans, vasculogenesis starts during the third week post-conception. Hemangioblastic cell cords differentiate *in situ* from placental mesenchymal stem cells in the villous cores. The cords elongate through proliferation and cell recruitment, and connect with the vasculature of the developing fetus. A feto-placental circulation starts around 8 weeks of gestation and perfusion of the placental villi by maternal blood occurs 4–6 weeks later. Elongation of the capillaries results in looping of the vessels. Obtrusion of both capillary loops and new sprouts results in the formation of terminal villi.

The placental vasculature continues to develop and adapt during pregnancy in response to the increasing requirements of the fetus. Reynolds *et al.,* [4] reported that the large increase in transplacental exchange, that supports the exponential increase in fetal growth during the last half of gestation, depends primarily on the dramatic growth of the placental vascular beds and the resultant large increases in uterine and umbilical blood flow [5]. The microvasculature of the placenta develops by the process of branching angiogensis that increases capillary numbers and surface densities. In the human placenta, branching angiogenesis, the formation of new vessels through sprouting, occurs from 5 weeks gestation through to 24 weeks gestation, while non-branching angiogenesis, the formation of capillary loops through elongation, predominates thereafter to term.

Available data supports the hypothesis that fetal growth and energy demand regulates placental growth and vascularization. For instance, the activity and expression of some nutrient transporters, in the mouse placenta, is modulated by fetal nutrient demands for growth [6]. Mutant mouse small placentae are capable of increasing glucose and amino acid transfer to meet the nutrient demands of the growing fetus, particularly in late gestation [6]. In human trophoblasts, System A, which includes the amino acid transport genes such as *Slc38A1, Slc38A2 and Slc38A4* has been shown to increase as pregnancy progresses, coinciding with increased fetal nutrient demands [7]. During late gestation in rat, glucose transporter genes *Slc2A3* and *Slc2A1* are upregulated and downregulated respectively, in placenta [6]. The molecular and cellular mechanisms that regulate vascularisation and angiogenesis within the placental villous tree during pregnancy remain to be fully elucidated. Previous studies have established the utility of rat and mouse models to eluciate the molecular and cellular mechanisms involved in placentation and placental development since it is more difficult to obtain human placenta at various gestational stages [8]–[10]. Genome-wide gene expression in rat placenta has been studied in late pregnancy [11] [12]. At embryonic day 18.5 (E18.5), RNA sequencing showed differential gene expression between the rat labyrinth zone, junctional zone and metrial gland [12]. At E21, Buffat *et al*. reported changes in genome-wide placental gene expression in rats exposed to an isocaloric low protein diet [11]. Goyal *et al.* (2010), studied global gene expression in placentae from two different rat species at 17.5 days of gestation and found 272 genes differentially expressed [13]. Large scale molecular analysis of the transcriptome of the normal rat placenta across mid to late gestation, however, has not been performed. In particular, gestational variation in the expresssion of genes known to be involved in angiogenesis within the rat placenta is yet to be documented.

The aim of this study, therefore, was to characterize gestational variation in rat placental gene expression using microarray analysis (22,000 gene probes) and specifically identify changes in the molecular pathways involved in angiogenesis. The hypothesis to be tested was that genes involved in regulating angiogenesis are differentially expressed during late gestation compared to late mid-gestation. Placentae were collected at four gestational ages (E14.25; E15.25; E17.25 and E20) and gene expression was compared.

## Materials and Methods

### Animals and Diets

All animal experiments were performed at the Department of Anatomy and Developmental Biology, Monash University (Melbourne, Australia) with the approval of The School of Biomedical Sciences Animal Ethics Committee of Monash University. Experiments were carried out in accordance with the National Health and Medical Research Council of Australia *“Australian Code of Practice for the Care and Use of Animals for Scientific Purposes”* (7th edition, 2004).

*Sprague Dawley* dams were allowed to adapt to the animal house for one week, consuming standard chow diet and water *ad libitum* prior to start of the study. The animals were maintained in a light controlled environment (12 h light/dark cycle) throughout this study and fed on a standard chow diet. Animals were time mated in a 3 hour period, with male *Sprague Dawley* rats. This was designated as Day 0 of pregnancy. The rationale of using a 3 h window mating time is to reduce variability of gestational age among the offspring and to maximize the accuracy in staging of gestation. Pregnancy was confirmed at the time of sacrifice. After mating, dams were housed individually. All animals had access to food and water *ad libitum* throughout this study.

### Tissue Collection

The pregnant dams were anaesthetized (Isoflurane Rhodia Australia P/L, VIC, Australia) and sacrificed at embryonic day (E) 14.25, 15.25, 17.25 and 20 (n = 6 per gestational age). Whole placentae were collected from the pregnant dams, weighed and then snap frozen in liquid nitrogen. Tissues were stored at −80°C until processed and analysed.

### RNA Isolation

Total RNA was extracted from 30 mg of pulverized frozen placental tissue (n = 6 placentae per gestational age group, one placenta per dam), using the AllPrep DNA/RNA Mini Kit (Qiagen) as per manufacturers’ instructions. Genomic DNA was removed by On-column Dnase1 treatment. Following extraction, total RNA was quantified via NanoDrop ND-1000 spectrophotometer (Thermo Scientific, DE, USA). RNA quality was verified using an Agilent 2100 Bioanalyzer (VIC, Australia) prior to the analysis. RNA samples that fulfilled the following criteria were selected for microarray analysis: (i) RIN >8.5; (ii) 260/280 ratio >2; (iii) 260∶230>1. RIN (RNA Integrity Number) values were greater than 8.7. For qPCR, the RNA was reverse transcribed using the QuantiTect Reverse transcription kit (Qiagen) using 1 µg of RNA per sample.

### Microarray Analysis on Illumina Rat Ref Arrays

For the microarray analysis, 500 ng of total RNA was converted to double stranded cDNA and this was used to generate biotinylated cRNA probes using the Illumina TotalPrep RNA Amplification Kit. Biotin-labelled cRNA were then hybridized to Illumina RatRef-12 Expression BeadChip (San Diego, CA, USA). Slides were scanned on a BeadStation 500 System using Beadscan software Version 3.5.31. No RNA samples were pooled in this analysis, each of the placental samples was analyzed independently. Samples were hybridized into wells at random. Two chips of 12 wells were used for the experiment. Illumina Whole-Genome Gene Expression BeadChips consist of oligonucleotides immobilized to beads held in microwells on the surface of an array substrate. Labelled sample cRNA were detected by hybridization to 50 mer probes on BeadChip. Post washing and staining, BeadChips were scanned on a BeadArray reader. Array experiment readout deposited on ArrayExpress. ArrayExpress Accession number is E-MTAB-1987.

### Quantitative Real-Time PCR Validation Experiments

qPCR was used to validate the expression of three placental genes (Ptgs2, Pla2g2a and Nos2) known to display gestational age changes in expression. Each experiment was performed in duplicate and normalized against the expression of β-Actin (Actb) using the ddCt method [14]. RNA was reverse transcribed into complentary DNA strands, using the Quantitect Reverse transcription kit (Qiagen, VIC, Australia) following manufacturers instructions. Primers used for qPCR throughout the study are detailed in Table S1 and were all designed using PRIMER-BLAST/ncbi against *Rattus Norvegicus* mRNA. qPCR analyses was performed using ABI 6000 using standard SYBR green from Life Technologies (VIC, Australia). Normalized Ct ratios were subjected to ANOVA. Spearman’s Rank Correlation, p<0.05 was used for correlation plots. Prior to statistical analysis of gene expression during gestation, homogeneity of variance and normality were assessed using Bartlett’s and Shapiro-Wilk tests, respectively. Normally distributed and homogenous data were assessed by ANOVA otherwise data were analysed using non-parametic Kruskal-Wallis tests.

### BioInformatic Anayses and Statistics

Bioinformatic analysis was performed by using the Illumina Beadstudio and Significance Analysis of Microarry (SAM, Stanford University) software. Data were normalized by performing a probe-intensity transformation and normalization via the Lumi package, Bioconductor. After normalization, differentially expressed placental genes (*i.e.* >2 fold expression, false discovery rate of <1%) were identified using SAM and were further analysed using Web-based Gene Set Analysis Toolkit (WebGestalt, http://bioinfo.vanderbilt.edu/webgestalt/). Genes were assigned to their respective functional classes based on the Gene Ontology (GO) database. Differences in group means were assessed by post-hoc comparisons (Bonferroni tests).

Normalized expression data were subjected to Principal Component Analyses (PCA, XLSTAT). 2D observation plots were generated for individual gestational age samples and correlation circles for the 31 angiogenic genes analyzed.

## Results

### Mircoarray Analysis

**Figure 1:**
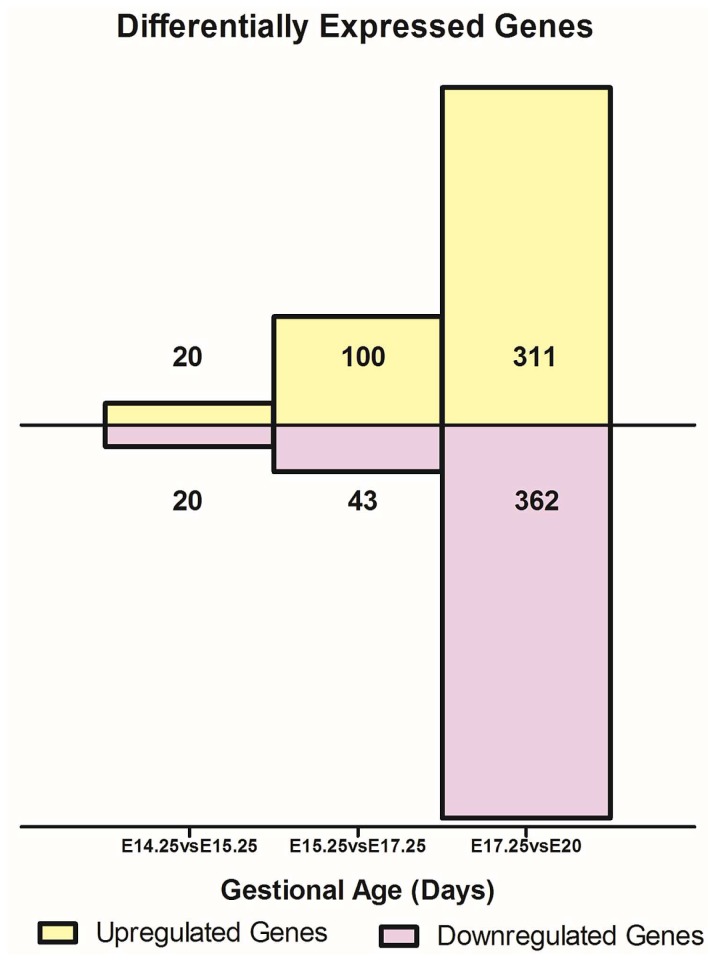
Differentially genes expression during gestation. **A)** The total number of differentially expressed genes that were up and down regulated between the 3 gestational groups *i.e.* E14.25 vs. E15.25, E15.25 vs. E17.25 and E17.25 vs. E20 are displayed as fold change (cut-off >2 and FDR<1%). **B)** Overall gene expression fold changes from the earliest time point under study E14.25 right up to E20.

**Figure 2:**
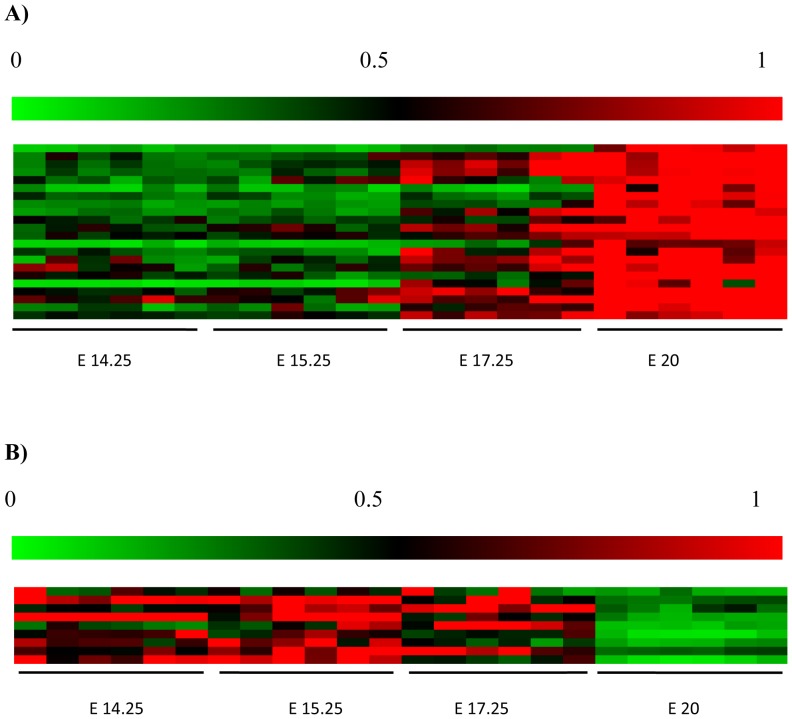
Heat Map of relative expression of Angiogenic genes. Of the 31 differentially expressed genes, 22 were up regulated (**A**) and 9 were down regulated (**B**). The heat map diplays data for 6 individual placentae for each gestational age. The genes correspond to the gene list in Table 1, where a change from green, black to red indicates and increase in signal. The colour legend above each map shows relative microarray signal ranges between 0 to 1. Each coloured rectangular box within the heat map represents a separate rat placenta sample. The samples are grouped according to gestational age *i.e.* E14.25, E15.25, E17.25, E 20.

To characterize gestational age dependent changes in gene expression, microarray analysis was performed on placental cDNA obtained from pregnant rats at gestational days E14.25, E15.25, E17.25 and E20. A cutoff of >2 fold change in expression and false discovery rate of less than 1% was used to ascribe the differential expression of genes. Between gestational age E14.25 and E15.25, 40 gene transcripts were differentially expressed, between E15.25 vs E17.25, 143 gene transcripts were differentially expressed and between E17.25 vs E20, 678 were differentially expressed using SAM Analyses (Figure 1). Specific differentially expressed angiogenic genes were then selected from the SAM readout and a heat map (Figure 2) displays expression changes over time.

### Quantitative RT-PCR Validation of Gestational-age Dependent Genes

**Figure 3:**
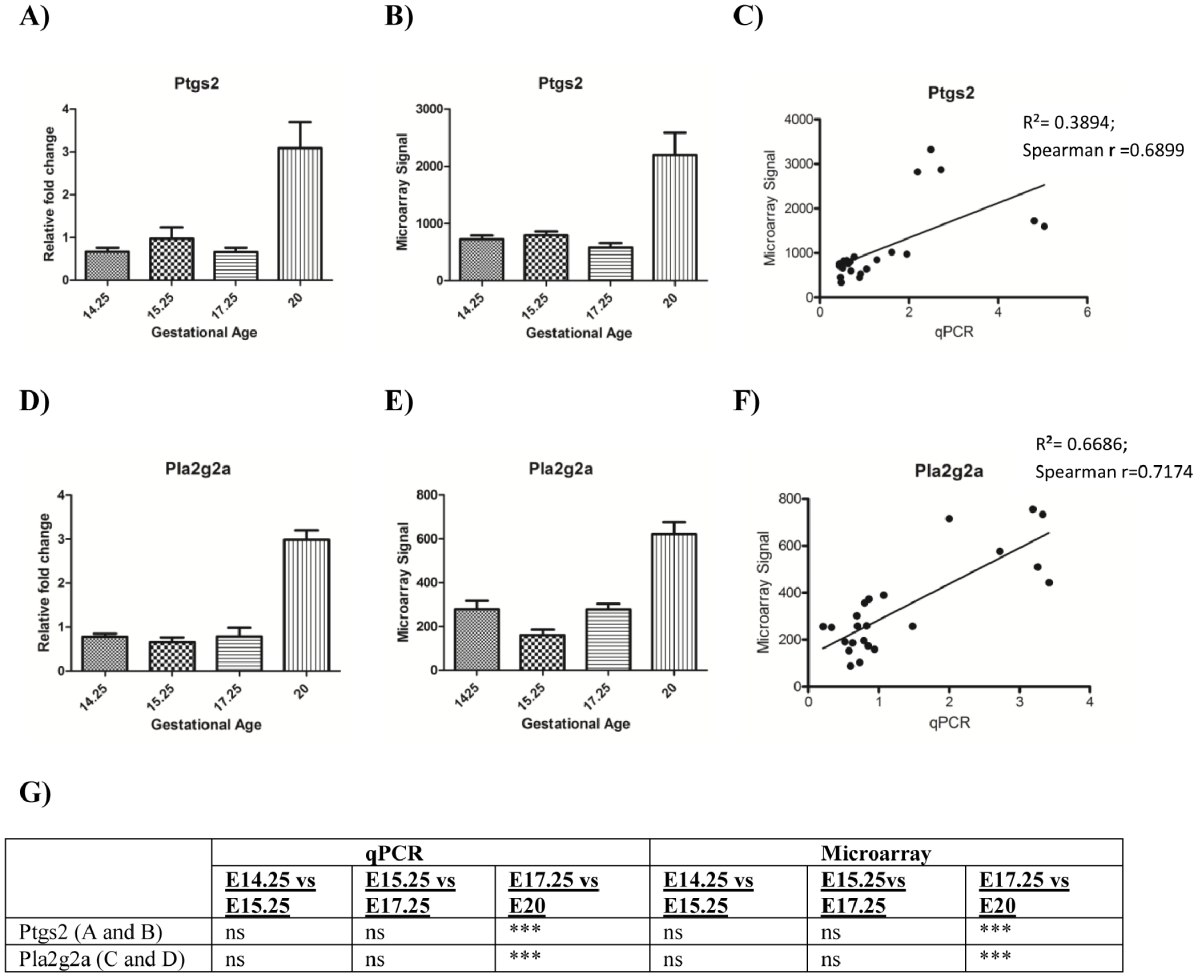
Comparison of qPCR and microarray expression data. qPCR and Microarray data for *Ptgs2* are shown in panels **A** and **B** respectively; statistical significance (p values) is presented in panel **G**. **C** represents linear correlation between qPCR and microarray data for *Ptgs2* (p<0.05). qPCR and microarray results for *Pla2g2a* are shown in panels **D** and **E** respectively; statistical significance (p values) are presented in panel **G**. Panel **F** indicates the linear correlation (Spearman’s rank correlation) between qPCR and microarray data (p<0.05). Both genes display late gestational increase. Where * = p<0.05, p<0.0005 = **; p<0.0001 = *** and ns = p>0.05 using one-way ANOVA and post-hoc tests (Bonferroni test).

**Figure 4:**
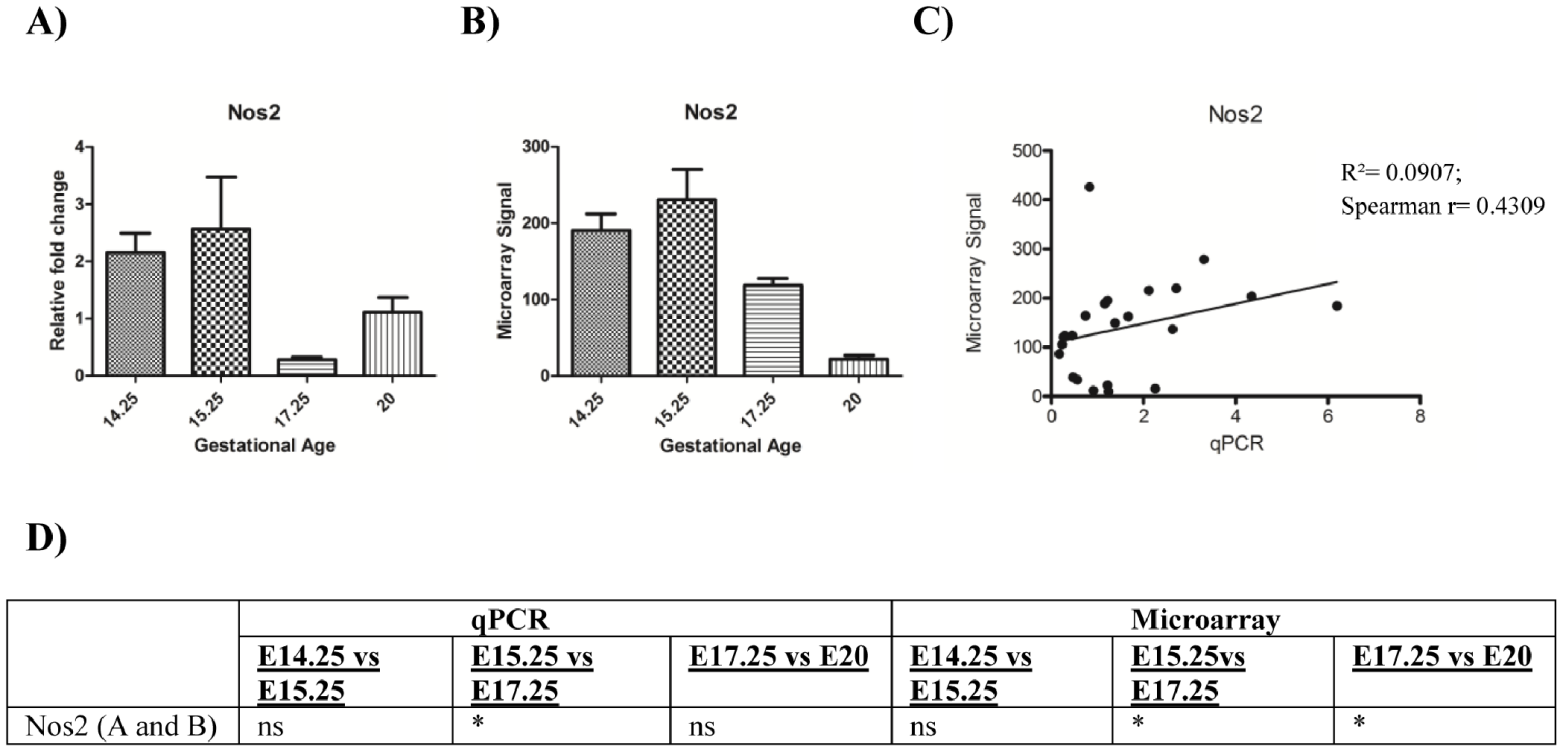
Comparison of qPCR and microarray data for Nos2. qPCR and microarray results of *Nos2* are presented in panels **A** and **B**, respectively. Statistical significance (p values) is presented in panel **D**. Panel **C** depicts linear correlation (Spearman’s rank correlation) between qPCR and microarray data (p<0.05) Both qPCR and microarray data display a significant decrease in expression with gestational age. (* = p<0.05, p<0.0005 = **; p<0.0001 = *** and ns = p>0.05).

qPCR data were consistent with microarray data and established that both *Ptgs2* and *Pla2g2a* remain relatively constant throughout E14.25, E15.25 and E17.25 and increase dramatically (and also consistent with literature) towards the end of gestation at E20 (Figure 3). *Nos2* on the other hand, is downregulated towards E17.25 and E20 (Figure 4 and Figure S2).

### Classification of Genes Involved in Blood Vessel Morphogenesis and Angiogenesis

Genes were classsified into pathways using GO (gene ontology) based upon their Entrez IDs. Within the *Blood Vessel Morphogenesis* and *Angiogenesis* pathway a total of 31 differentially expressed genes were identified (GO:0048514 : blood vessel morphogenesis [412 gene products] and GO:0001525 : angiogenesis [333 gene products]). 22 of these genes displayed increased expression and 9 genes decreased expression across the gestational age groups. Between E14.25 and E15.25 none of the 31 genes were differentially expressed, between E15.25 and E17.25, 15 genes were differentially expressed and between E17.25 and E20, 26 genes were differentially expressed (Table 1, Figure 2, Figure S1 and S2). Figure S4 displays the mean centroid analyses of A) upregulated and B) downregulated genes.

**Table 1:** List of Angiogenic genes identified and pairwise comparison of gene expression between gestational age groups.

Gene Identifiers		E14.25 vs E15.25		E15.25 vs E17.25		E17.25 vs E20		E14.25 vs E20	
Gene name	Entrez ID	Fold change	p value	Fold change	p value	Fold change	p value	Fold change	p value
**Up- regulated** **22 genes**									
**Bmp4**	**25296**	–	ns	–	ns	3.8	***	6.77	***
**Vegfa**	**83785**	–	ns	<2	*	–	ns	3.04	***
**Mmp14**	**81707**	–	ns	<2	***	–	ns	3.44	***
**Acvrl1**	**25237**	–	ns	2.8	***	–	ns	3.42	***
**Edn1**	**24323**	–	ns	–	ns	<2	***	3.61	***
**IL18**	**29197**	–	ns	–	ns	6.4	***	6.74	***
**Gpx1**	**24404**	–	ns	–	ns	3.7	***	3.69	***
**Id1**	**25261**	–	ns	–	ns	<2	***	4.27	***
**Cd36**	**29184**	–	ns	2.8	***	<2	***	4.87	***
**Hs6st1**	**316325**	–	ns	–	ns	<2	***	<2	***
**Emcn**	**295490**	–	ns	<2	**	<2	***	2.94	***
**Rhob**	**64373**	–	ns	–	ns	<2	***	2.31	***
**Prl7d1**	**84377**	–	ns	–	ns	<2	***	9.29	***
**Angptl4**	**362850**	–	ns	3.1	***	3.7	***	6.93	***
**Sox18**	**311723**	–	ns	<2	**	–	ns	<2	***
**Klf5**	**84410**	–	ns	<2	*	<2	**	<2	***
**Pf4**	**360918**	–	ns	–	ns	3.0	***	2.38	***
**Cav1**	**25404**	–	ns	6.7	***	<2	***	15.46	***
**Sgpl1**	**286896**	–	ns	–	ns	<2	***	2.64	***
**Notch4**	**406162**	–	ns	–	ns	<2	***	<2	***
**Tek**	**89804**	–	ns	<2	*	2.2	***	4.72	***
**Tgm2**	**56083**	–	ns	<2	**	<2	***	2.34	***
**Down-regulated** **9 genes**									
**Itgb2**	**309684**	–	ns	–	ns	>0.5	**	>0.5	***
**ItgaV**	**296456**	–	ns	–	ns	>0.5	*	0.41	***
**Itga4**	**311144**	–	ns	>0.5	*	0.4	***	ns	***
**Foxm1**	**58921**	–	ns	>0.5	**	0.4	***	0.20	***
**Anpep**	**81641**	–	ns	–	ns	0.2	***	0.2	***
**Gbx2**	**114500**	–	ns	–	ns	0.2	***	0.13	***
**Atg5**	**365601**	–	ns	>0.5	**	–	ns	>0.5	***
**Cited2**	**114490**	–	ns	–	ns	0.5	***	0.5	***
Nos2	24599	–	ns	>0.5	*	0.2	***	0.16	***

E14.25 vs E15.25 - no significant differences in gene expression. E15.25 vs E17.25–15 of 31 genes were differentially expressed. E17.25 vs E20–26 of 31 genes were differentially expressed. E14.25 vs E20 shows gene expression changes for each gene from E14.25 vs E20. Significance was ascribed where gene expression was changed by <2 (* = p<0.05, p<0.0005 = **; p<0.0001 = *** and ns = p>0.05). The order of the genes listed in the table corresponds to the Heat Map on Figure 2.

### Quantitative RT-PCR of Angiogenic Genes

**Figure 5:**
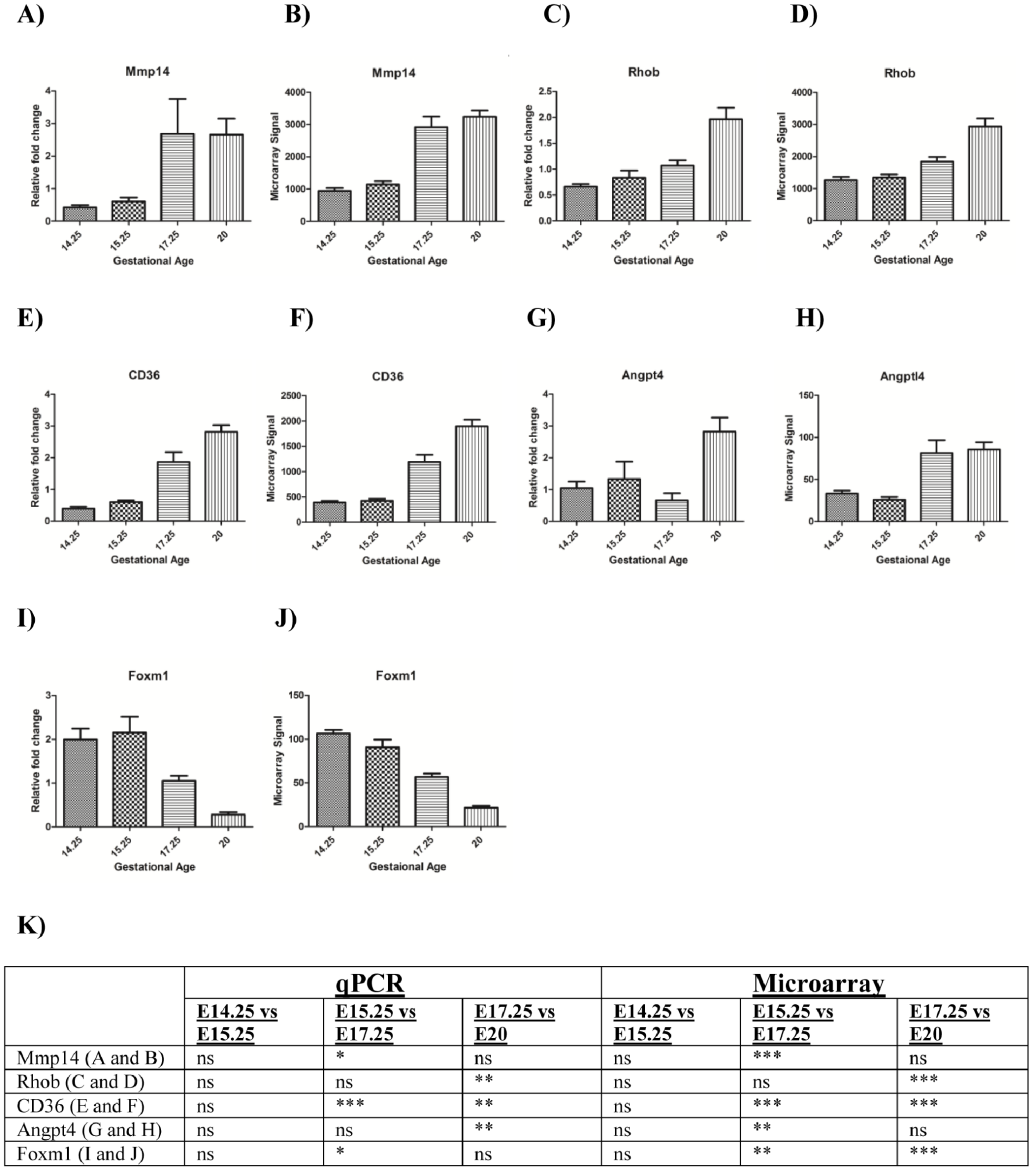
qPCR validation of 5 Angiogenic genes. For *Mmp14*, qPCR are microarray data are presented in panels **A** and **B**, respectively; for *Rhob* in panels **C** and **D**; for *CD36* in panels **E** and **F**; for *Angpt4* in panels **G** and **H**; for Foxm1 in panels **I** and **J**. Statistical significance (p values) is presented in panel **K**. Where p<0.05 = *; p<0.0005 = **; p<0.0001 = *** as assessed by one way ANOVA and post-hoc tests (Bonferroni test). The x-axes in all graphs represent the 4 gestational age groups. Y-axes for qPCR graphs denote relative fold change normalised to β-actin. The y-axes for the microarray bar charts denote microarray hybridisation signals.

The expression of five angiogenic genes (*Mmp14*, *CD36, Angpt4*, *Rhob* and *Foxm1)* was confirmed by qPCR (Figure 5). For all genes examined, a significant correlation between microarray and qPCR data was established (Spearman’s Rank Correlation, p<0.05).

### Principal Component Analysis

**Figure 6:**
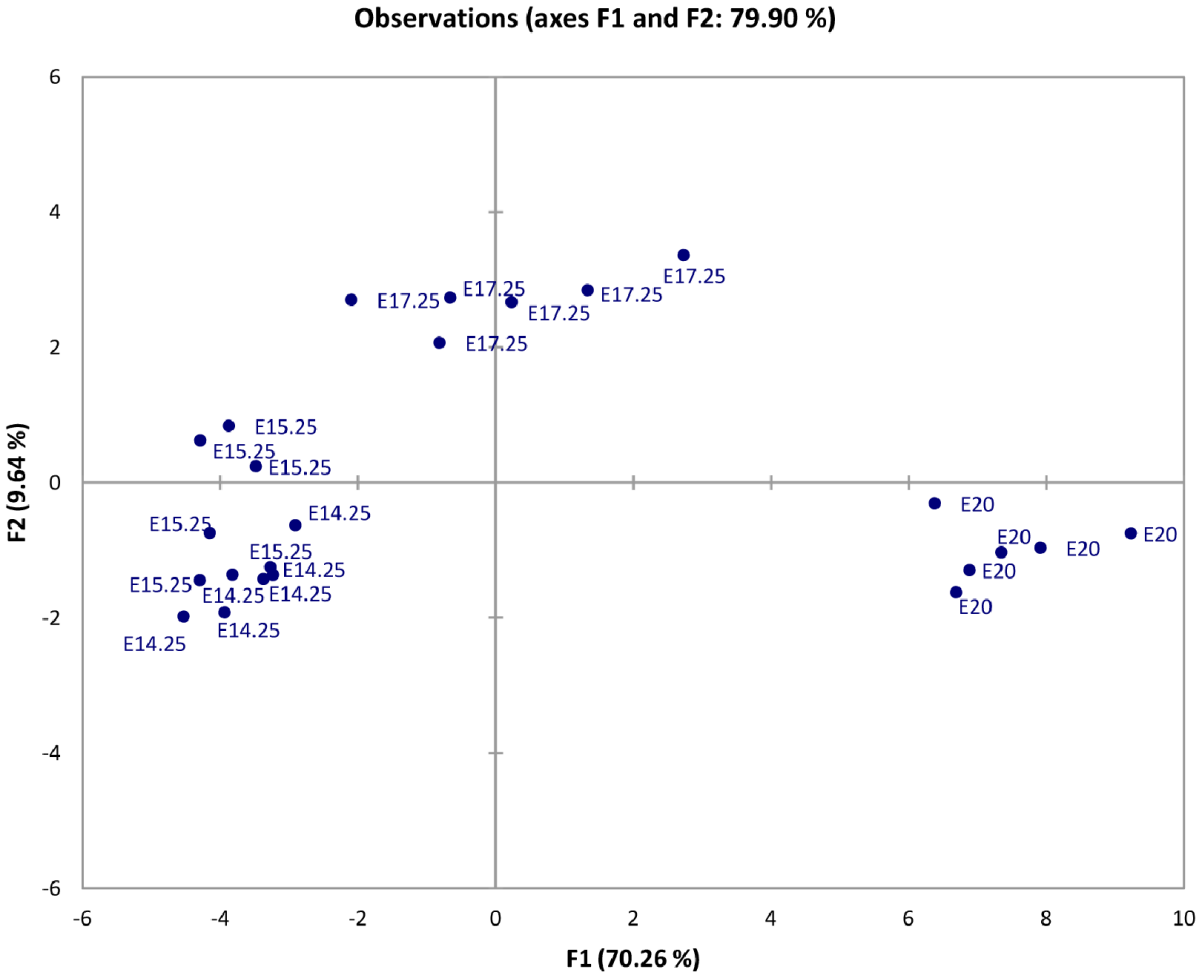
Principal Component Analysis (PCA). Principal components F1 *vs*. F2 for individual gestational age groups (n = 6, E14.25, E15.25, E17.25 and E20) for all 31 angiogenic genes are plotted to identify variation in gene expression. Day E14.25 and E15.25 samples cluster together, indicating a similar genes expression profile between these 2 groups. Samples within the E17.25 and E20 groups, however, display independent clustering, indicative of significantly altered gene expression profiles. Biological replicates within each gestational age group are tightly clustered.

PCA of placental angiogenic gene expression separated data into three distinct groups. E14.25 and E15.25 samples clustered together, while E17.25 and E20 samples partitioned independently (with F1 accounting for up to 70% of sample variation, Figure 6). These data are consistent with the hypothesis that the expression profile of *Blood Vessel Morphogenesis and Angiogenesis* genes in the rat placenta changes significantly after 70% of gestation is reached and continues to change thoughout the remainder of gestation. Gestational age based PCA analyses are presented in Figure S3. Furthermore, PCA indicates that the biological variation within the gestational age category for these genes is small (n = 6). This conclusion was additionally supported by ANOVA where the variance in microarray data was partitioned between, gene (p<0.00001), gestational age (p<0.00001) and biological replicates (p>0.9).

## Discussion

The aim of this study was to identify mid-late gestation changes in the expression of genes in the whole rat placenta using microarray analysis. In particular, this study focused on changes in genes involved in angiogenic pathways. During mid-late gestation, fetal demand for energy substrates and gas exchange increases dramatically in the absence of a concomitant increase in placental mass. An increase in branching angiogenesis contributes to placental accommodation during this later gestational period. The data obtained establish that the expression of approximately 7% of genes profiled (22,000) within the placenta change significantly between E14.25 and E20 days of gestation. Of this 7%, 42% of genes increased expression and 58% decreased expression. A subset of 31 genes known to be involved in the *Blood Vessel Morphogenesis* pathways were identified, of which ∼70% displayed increased expression with gestational age. RNA sequencing of different rat placental regions at E18.5 showed that the blood vessel development pathway was prominent in genes that were differentially expressed in the labyrinth zone versus the junctional zone and the metrial gland [12]. In the metrial gland, the vascular development pathway was enriched [12]. These results indicate that the vasculature in the rat placenta is developing late into gestation.

The microarray expression data for eight genes were orthogonally validated by qPCR. These genes included three genes previously known to display gestational age and labour-associated changes and five genes identified in this study that displayed altered expression during gestation. Previously, we and others, have identified late gestation and pre-labour changes in the expression of enzymes involved in the biosynthesis of eicosanoids (*i.e.* prostaglandin synthase 2, *Ptgs2* ([15]–[18] and phospholipase A_2_ (*Pla2a)* [19]) and in nitric oxide (*i.e*. inducible nitric oxide synthase, *Nos2* [20]). Consistent with these observations, the gene expression of prostaglandin synthase 2 and secretory *Pla2a* increased (from E17.25 to E20) and *Nos2* decreased (from E15.25 to E20) during gestation (as determined by both microarray analysis and real-time PCR). This finding is consistent with current literature. Furthermore, the data for *Ptgs2* are consistent with the literature where expression is known to increase prior to the onset of labour [10], [12], [13]). Phospholipases hydrolyze phospholipids into fatty acids. Phospholipase A2 is an important mediator of arachidonic acid formation, which are the substrates of eicosanoids such as prostaglandins. It has been reported that there is a relative abundance of phospholipase protein in late pregnancy [19].

Of the 31 *Blood Vessel Morphogenesis* pathway genes that displayed differental expression during gestation in this study, the expression of *Cd36, Mmp14, Angpt4, Rhob* and *Foxm1* was confirmed by qPCR on same samples. In all cases, concordance between microarray and qPCR data was confirmed. These five genes were chosen on the basis of their function and the fold changes shown in Table 1.

CD36 is a, cell membrane scavenger receptor involved in angiogenesis, inflammation and lipid metabolism [21]. In microvascular endothelial cells it functions as a receptor for Thrombospondin-1 (TSP-1) [22]. CD36 has been implicated in inflammatory processes via the activation of phospholipases and the formation of prostaglandins [23] and the cellular uptake of fatty acids via the activation of peroxisome proliferator–activated receptor γ [24]. In this study, *Cd36* expression increased by 2.8 fold during E15.25 to E17.25 days of gestation and remains significantly elevated till labour onset. Interestingly, increased expression of *Cd36* mRNA and protein is observed in cord blood from late pregnancy compared with early pregnancy samples.

*Mmp14* encodes for MMP14 (EC 3.4.24.80, also known as MT1-MMP) which is a key extracellular matrix-remodeling enzyme that promotes collagen remodeling. MMP14 is a dominant cell-surface protease required for endothelial cell tube morphogenesis, invasion and for the creation of vascular guidance tube [23]. However, there is only limited information about the specific role of MMP14 role in placental angiogenesis. In mouse placentae, MMP14 is strongly expressed in the labyrinth region [25]. This region of the placenta is critical for the formation of syncytiotrophoblast and the subsequent formation of fetal vessels. In human placentae, immunoreactive MMP14 is expressed in term and preterm syncytiotrophoblast cells, where it may contribute to the cleavage and release of endoglin [26] and in the endothelium of feto-placental vessels. Endoglin has been suggested to be a major factor in the development of preeclampsia [27]. Placental expression of MMP14 is greater in pregnancies complicated by gestational diabetes mellitus [28] and, *in vitro* it is induced by insulin and IGF-II *in vitro* [28] and HIF-1α [24]. In this study, Mmp14 mRNA expression increased from E15.25 to E17.25 (the peak of trophoblast invasion takes place at this stage) [29]. Another gene that is involved in prostaglandin regulation is *Sgpl1* (SGPL1, EC 4.1.2.27) which catalizes the irreversible degradation of the sphingosine-1-phosphate (S1P) [30]. S1P is a bioactive lipid mediator that promotes cell proliferation, survival, migration, adherence, inflammation and angiogenesis [31]. SGPL1, thus, regulates the available pool of S1P and inhibits its signalling activities. SGPL1 may regulate S1P-induced Ptgs2 expression in the rat myometrium [32]. In this study, *Sgpl1* expression increased 2.6 fold during gestation from E14.25 to E20.

Angiopoeitin like Protein 4 (EC 2.6.1.2) expression was low at E14.25 and E15.25 and increases at E17.25 remaining constant till E20. Over the gestational period assessed in this study, *Angpt4* gene expression increased ∼7-fold. *Angpt4*, like the other angiopoietins 1 and 2 has been reported to play a role in angiogenesis [33]. Yamakawa *et al.,* reported that ANGPT4 increased endothelial cell migration and tube formation *in vitro* and reduced vascular leakage [34]. Angiogenic effects of ANGPT4 have also been observed in glioblastoma [35]. To date little is known about the biological functions of ANGPT4 in the placenta. However, ANGPT1 has been shown to potentiate VEGF activity and work together to increase the luminal diameter of blood vessels in sheep placenta [36]. In this study, the receptor for ANGPT1, ANGPT2 [36] and ANGPT4, TEK (*Tek*) showed an increase in placental expression during from E15.25 to E17.25 and further from E17.25 to E20 (fold change 2.2) of gestation. TEK has been implicated in the regulation of angiogenesis and cell proliferation, migration and survival [36]. *Tek* expression was significantly, positively correlated (p<0.05) with *Angpt4* expression during gestation, (upregulated prior to labour) indicating the possibility that they are working together to promote late gestational angiogenesis. TIE 1 is the other receptor for ANGPT4 and in our Microarray study, interestingly, *Tie1* expression does not change from E14.25 right up to E20 (Data not shown).

Ras homolog family member B, *Rhob* has not been extensively studied in the placenta. The data obtained in this study (both microarray and qPCR) establish that *Rhob* expression gradually increases during gestation (from E14.25 to E20), by a fold change of 2.3. Recently, RHOB (EC 3.6.1.47) was shown to regulate endothelial cell migration, sprouting, and capillary morphogenesis [37], although the mechanism by which RHOB regulates angiogenesis is not well understood. Howe and Addison (2012) concluded that RHOB plays a significant role in VEGF-induced endothelial cell morphogenesis, in part, by negatively modulating the activity of RHOA, [37]. Both RHOA (Ras homolog family member A) and RHOB are essential downstream effectors of VEGF signalling in the angiogenic process. siRNA-inhibition of *RhoB* results in increased RHOA activation in response to VEGF (vascular edothelial growth factor) stimulation [37]. *Vegf* was also seen to upregulate the expression of *RhoB* [26]. In our study, we see that both *RhoB and Vegf* are up regulated towards late gestation, first *Vegf* expression increases at E15.25 and then *Rhob* expression increases downstream at E17.25, indicating a positive correlation. Placental expression of *Vegfa* was increased 3-fold between E14.25 and E20 in the present study. VEGF is known to be an important regulator of angiogenesis in placenta [38], [39] and its increased expression during late gestation has been previously reported [5], while its receptor *Flk* (i.e *VegfR2*) is downregulated from E14.25 vs E20, prior to labour (Microarray data not shown).NOTCH-4 is a modulator of angiogenesis in human placenta and regulates placental cell fate [40]–[42]. Similar to RHOB expression, Notch signalling acts downstream of VEGF. Notch signalling helps to regulate endothelial cell morphogenesis via activation of MMPs [43]. In this study, *Notch-4* expression doubled between E15.25 and E20, where its expression is elevated prior to term/labour.

The transcriptional regulator Foxheadbox M1 (*Foxm1*) was expressed during late midgestation (E14.25–E15.25, as assessed by both microarray and qPCR) but decreased 5-fold by E20. Little, however, is known about FOXM1 function in angiogenesis in the placenta. Interestingly, the onset of labour in humans is associated with decreased placental expression of Foxheadbox O4, (both *FoxO4* mRNA and protein) where it may function as a negative regulator of *Ptgs2* expression and prostaglandin biosynthesis [44]. In cancer development and progression, FOXM1 has been implicated in regulating the expression of the human endothelial cell caveolae-marker CAV-1 and *Foxm1* expression has also been linked to growth of glioma cells in tumour angiogenesis [45], [46]. Placental *Cav-1* mRNA expression increased during gestation in this study. Caveolin has been reported to facilitate VEGF/NO-mediated angiogenesis [47]. Our data show, *Cav-1* expression increased 6.7 fold between E15.25 and E17.25 and further increased till E20. CAV-1 is known to interact with FGFR1which modulates FGF2 related placental angiogenesis [48]. Unlike in cancer progression we show that an increase in *Cav-1 and Vegf* expression is negatively correlated with *Foxm1* expression. An increase in FOXM1 could have a negative effect on CAV-1 expression or its expression is perhaps acutely switched off prior to labour onset. Figure S5 shows CAV-1 protein localised intensively around placental blood vessels indicating its important role in Angiogenesis.

Angiogenic genes of significance that were upregulated (based on microarray data alone) during gestation included: bone morphogenetic protein 4 (*Bmp4*); transglutaminase (*Tgm* EC 2.3.2.13); sphingosine-1 phosphate lyase (*Sgpl1,* EC 4.1.2.27); endomucin (*Emcn*); Interleukin-18 (*IL-18*) and glutathione peroxidase 1 (*Gpx1*, EC 1.11.1.9).

Microarray analysis also identifed a suite of *Blood Vessel Morphogenesis* pathway genes that were downregulated during late gestation, prior to labor onset. These included, *Itga4, Itgb2, Anpep* and *Cited2*. The integrin subunits alpha 4 (*Itga4*) and beta 2 (*Itgb2*) have been previously identified in placentae [26], [28] [49]. ITGA4 forms a heterodimer with integrin beta 1 to function as a receptor for collagen. Increased expression of ITGA4 has been implicated in inhibition of endothelial cell migation during angiogeneis [49]. In this study, *Itga4* gene expression decreases during gestation (from E15.25 right up to E20) and Itgb2 expression decreases acutely prior to labour onset. *Cited2* gene expression also decreases during late gestation from E17.25 to E20 by two fold. In the mouse placenta, lack of CITED2 is characterized by disorganization of the placental fetal vasculature and a fewer trophoblast giant cells, spongiotrophoblasts and glycogen cells [50]. Hence *Cited2* is important for normal placental vascularisation.

Additionaly, during late gestation in rat, glucose transporter genes *Slc2A3* and *Slc2A1* are upregulated and downregulated respectively, in placenta [6]. This is consistent with our microarray data where *Slc2A3* expression increases from E15.25 to E17.25 and further increases at E20 and *Slc2A1* is increased from E15.25 to E17.25 and then gets downregulated at E20 (data not shown). Amino acid transport genes such as *Slc38A1, Slc38A2 and Slc38A4* have been shown to increase with gestation, coinciding with increased fetal nutrient demands [7], which is also consistent with our microarray data. We can relate the Angiogenic expression changes in this study to the expression of several transporter genes. In conclusion, the data obtained in this study extends our understanding of placental genes that may contribute to placental vascular accommodation during mid-late gestation and late-gestation, in particular, those involved in regulating placental angiogenesis. Several of these gene products work together either directly or indirectly. However, we are not able to ascertain that the pre labour associated changes seen in these 32 angiogenic genes, has any link with the actual labour process, even though some of the changes are acute. Further studies need to be carried out to prove a link between some or all of these genes with the onset or trigger of labour. The study identified gestational age-dependent changes in placental gene expression within the *Blood Vessel Morphogenesis* pathways that have not previously been characterized in rat placenta. Furthermore, it confirms and extends data for genes previously reported to display gestational and potential labor-associated changes. We also observed that, most expression changes for these genes, occurred between E17.25 and E20, prior to labour onset. One caveat that must be considered in interpreting the results obtained in this study is that mRNA was extracted from whole placental tissue. Therefore, it is not possible to attribute observed changes in gene expression to specific cell types that comprise the rat placenta. Nevertheless, the data obtained identify lead candidate genes for subsequent cell specific analyses (*e.g.* based cell selection using laser capture microscopy) and provide opportunity to further elucidate functional pathways that may be of significance in mid-late placental function in both normal and pathological pregnancies.

## Supporting Information

Figure S1 **Gestational Variation in Angiogenic gene expression of 22 genes that display a late gestational increase.** The x-axis indicates the gestational ages whereas the y-axis indicates the signal obtained from the microarray hybridisation using the Illumina Bead reader. These 22 genes had SAM p values <0.0001.
(TIF)

Figure S2 **Gestational Variation in Angiogenic gene expression of 9 genes that display a late gestational decrease.** The x-axis indicates the gestational ages in days whereas the y-axis indicates the signal obtained from the microarray hybridisation using the Illumina Bead reader. SAM p values were <0.0001.
(TIF)

Figure S3 **Two Dimensional PCA Observational plots for Gestational Age Comparison Groups:** A) E14.25 vs. E15.25. PCA plot F1 vs. F2. B) E15.25 vs. E17.25. PCA plot F1 vs. F2. C) E17.25 vs E20. PCA plot F1 vs. F2. Data were analyzed using Pearson Correlation Matrix (XLSTAT, p<0.05).
(TIF)

Figure S4 **Mean Centroid of Angiogenesis.** A) for upregulated genes B) for down regulated genes using Kruskal-Wallist test of Mean Centroid. A)E20 vs E14.25 = ***, E20 vs E15.25 = *** and E20 vs E17.25 = ns and B)E20 vs E14.25 = *, E20 vs E15.25 = *** and E20 vs E17.25 = ns. (* = p<0.05, p<0.0005 = **; p<0.0001 = *** and ns = p>0.05).
(TIF)

Figure S5 **Immunohistochemical Localization of CAV-1 and BMP4.** Intense staining for CAV-1 around placental blood vessels (**A** and **B**), BMP4 (**C** and **D**) in endothelial cells lining blood vessels and Rabbit IgG Isotype Negative Control (**E**). Haemotoxylin staining of nuclei shown in blue.
(TIF)

Table S1 **List of Primer oligonucleotide sequences used for qPCR experiments.**
(TIF)
