# Supplementary figures and images for: The Effect of Gestational Age on Angiogenic Gene Expression in the Rat Placenta
Source: PLoS One. 2013 Dec 31;8(12):e83762. doi: 10.1371/journal.pone.0083762 (PMC3877080; doi:10.1371/journal.pone.0083762)

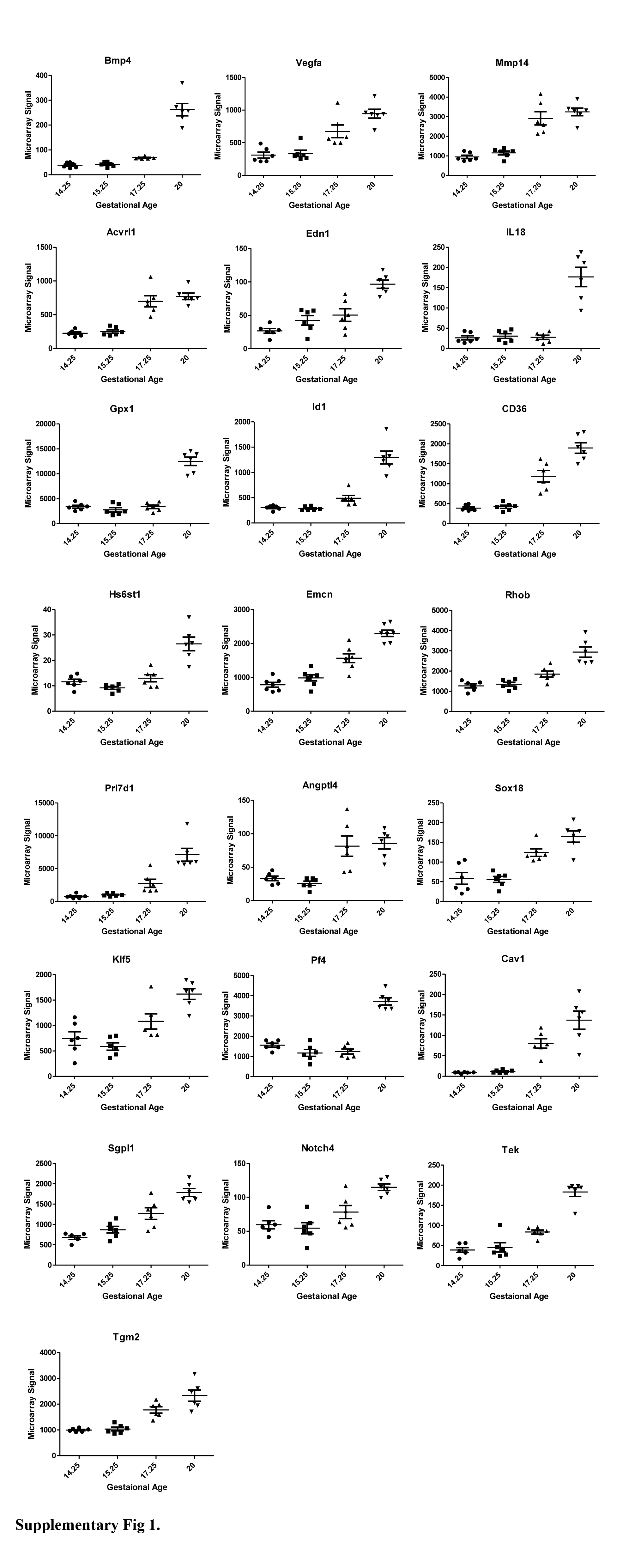

Supplement: Figure S1 — Gestational Variation in Angiogenic gene expression of 22 genes that display a late gestational increase. The x-axis indicates the gestational ages whereas the y-axis indicates the signal obtained from the microarray hybridisation using the Illumina Bead reader. These 22 genes had SAM p values <0.0001. (TIF) [file pone.0083762.s001.tif]

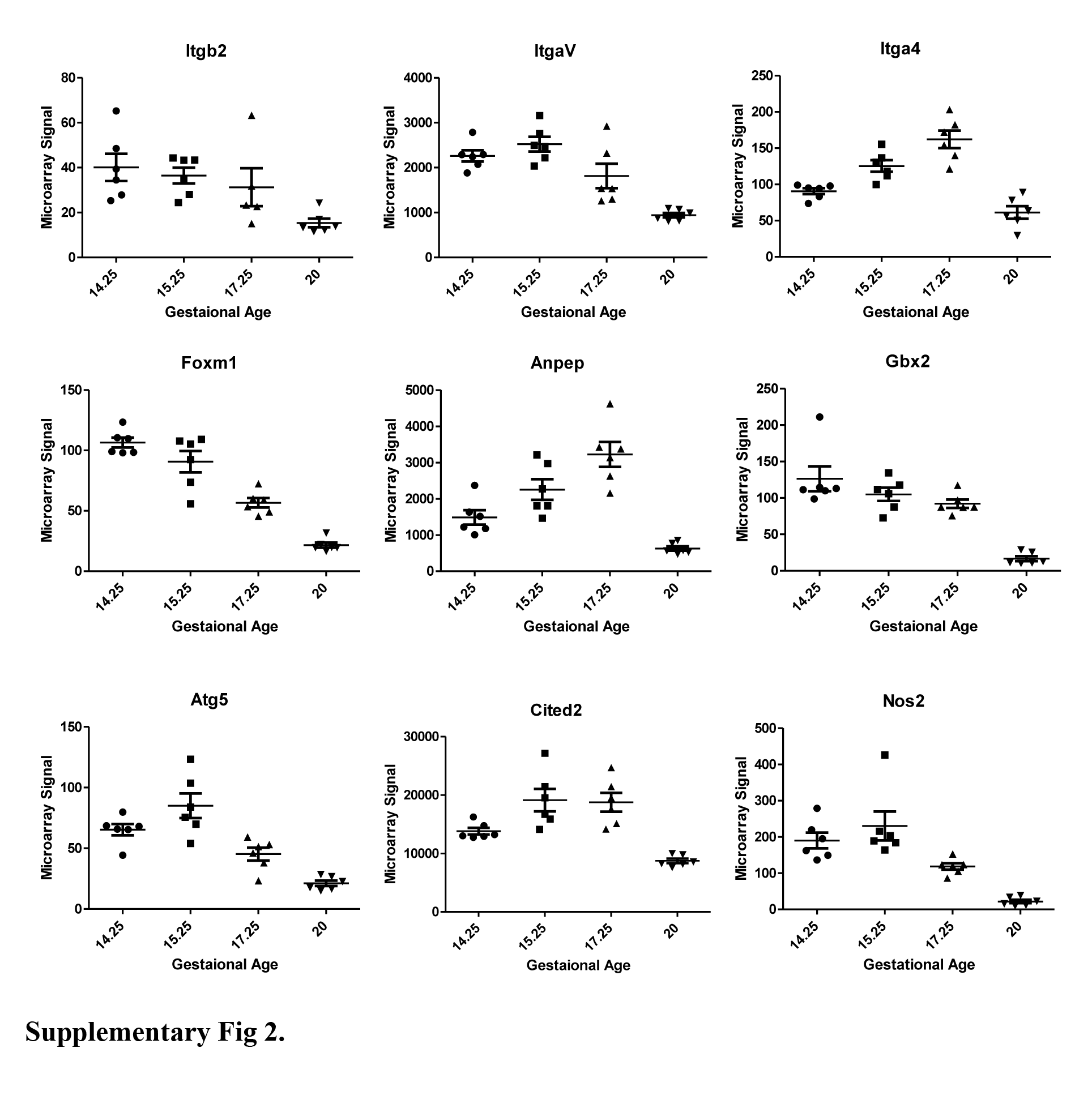

Supplement: Figure S2 — Gestational Variation in Angiogenic gene expression of 9 genes that display a late gestational decrease. The x-axis indicates the gestational ages in days whereas the y-axis indicates the signal obtained from the microarray hybridisation using the Illumina Bead reader. SAM p values were <0.0001. (TIF) [file pone.0083762.s002.tif]

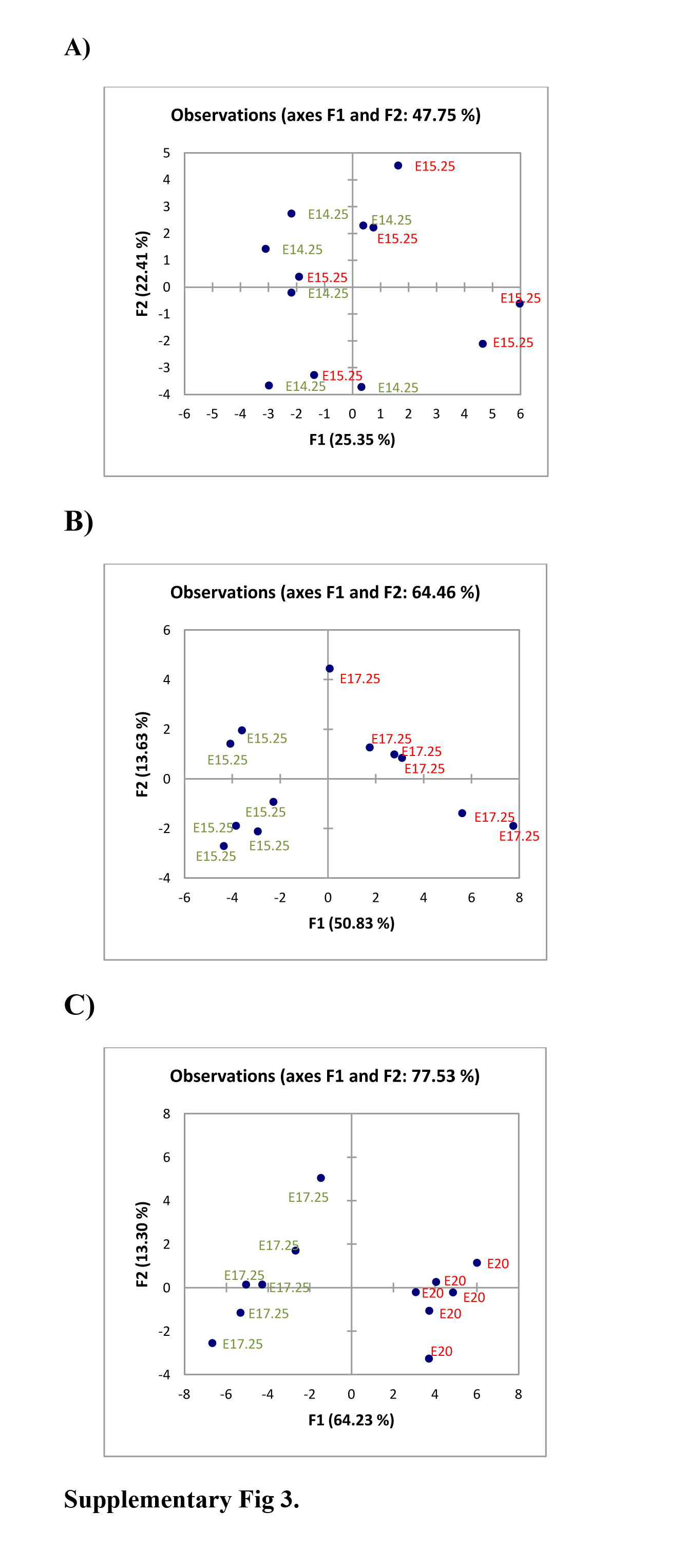

Supplement: Figure S3 — Two Dimensional PCA Observational plots for Gestational Age Comparison Groups: A) E14.25 vs. E15.25. PCA plot F1 vs. F2. B) E15.25 vs. E17.25. PCA plot F1 vs. F2. C) E17.25 vs E20. PCA plot F1 vs. F2. Data were analyzed using Pearson Correlation Matrix (XLSTAT, p<0.05). (TIF) [file pone.0083762.s003.tif]

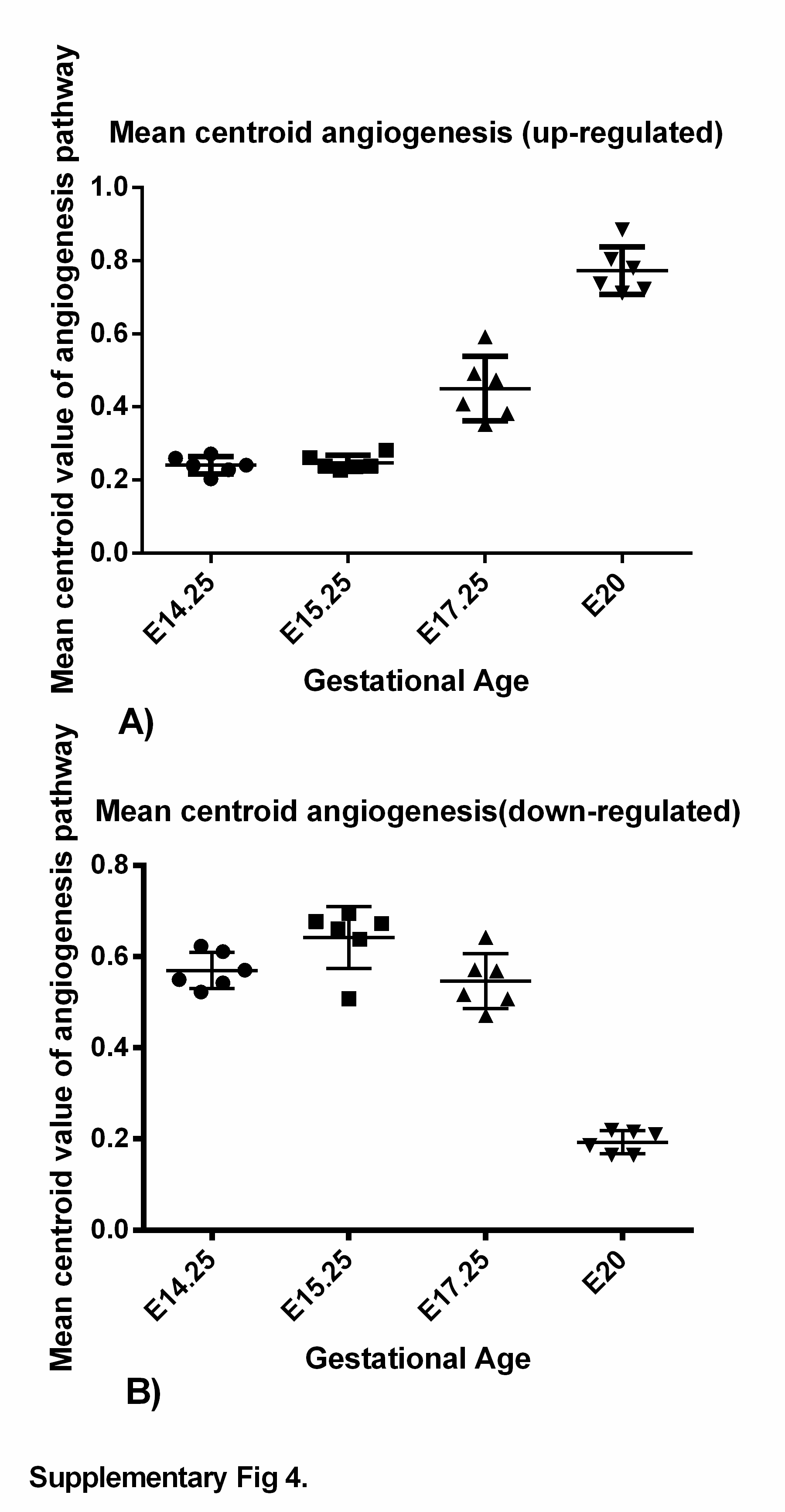

Supplement: Figure S4 — Mean Centroid of Angiogenesis. A) for upregulated genes B) for down regulated genes using Kruskal-Wallist test of Mean Centroid. A)E20 vs E14.25 = ***, E20 vs E15.25 = *** and E20 vs E17.25 = ns and B)E20 vs E14.25 = *, E20 vs E15.25 = *** and E20 vs E17.25 = ns. (* = p<0.05, p<0.0005 = **; p<0.0001 = *** and ns = p>0.05). (TIF) [file pone.0083762.s004.tif]

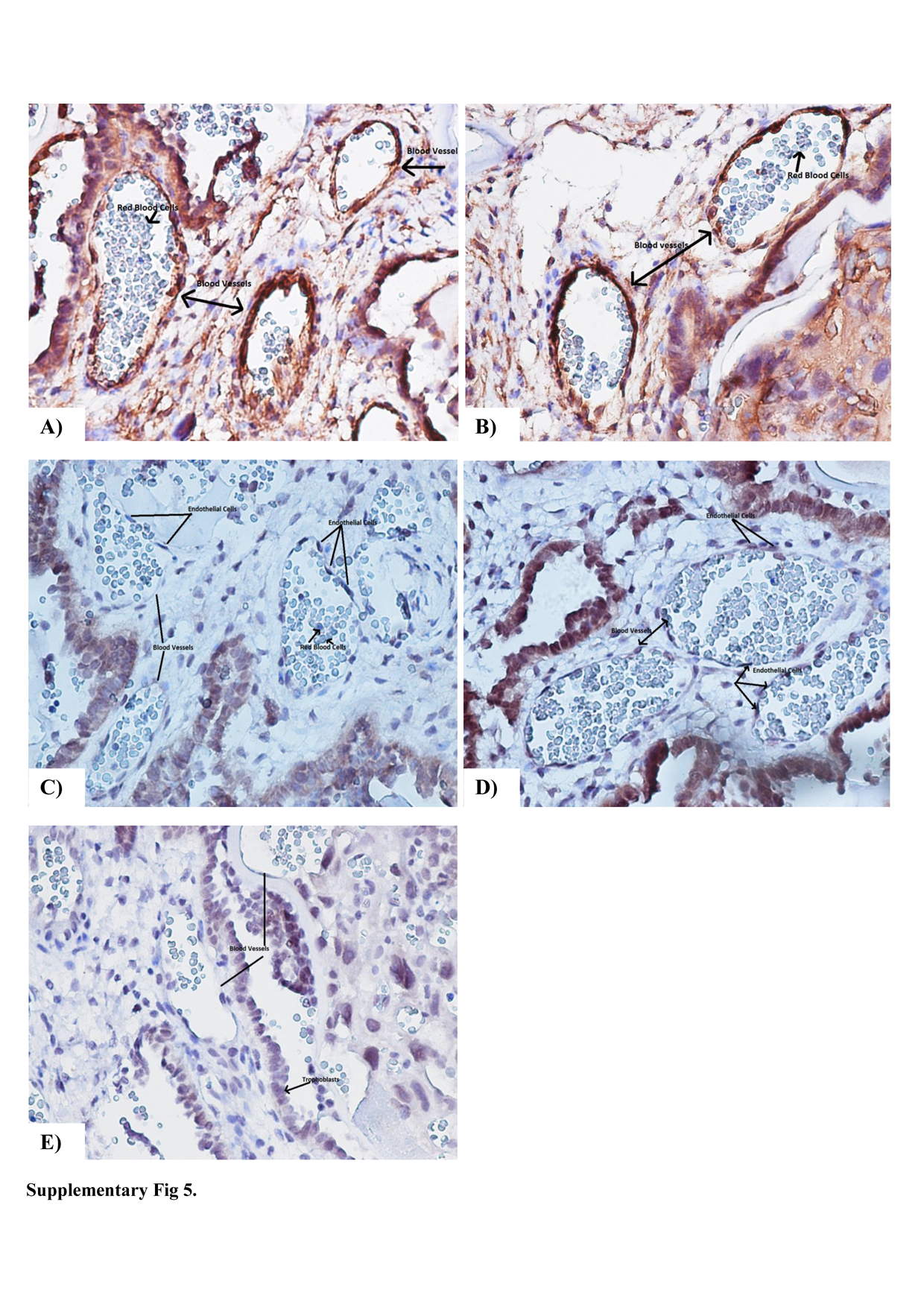

Supplement: Figure S5 — Immunohistochemical Localization of CAV-1 and BMP4. Intense staining for CAV-1 around placental blood vessels (A and B), BMP4 (C and D) in endothelial cells lining blood vessels and Rabbit IgG Isotype Negative Control (E). Haemotoxylin staining of nuclei shown in blue. (TIF) [file pone.0083762.s005.tif]

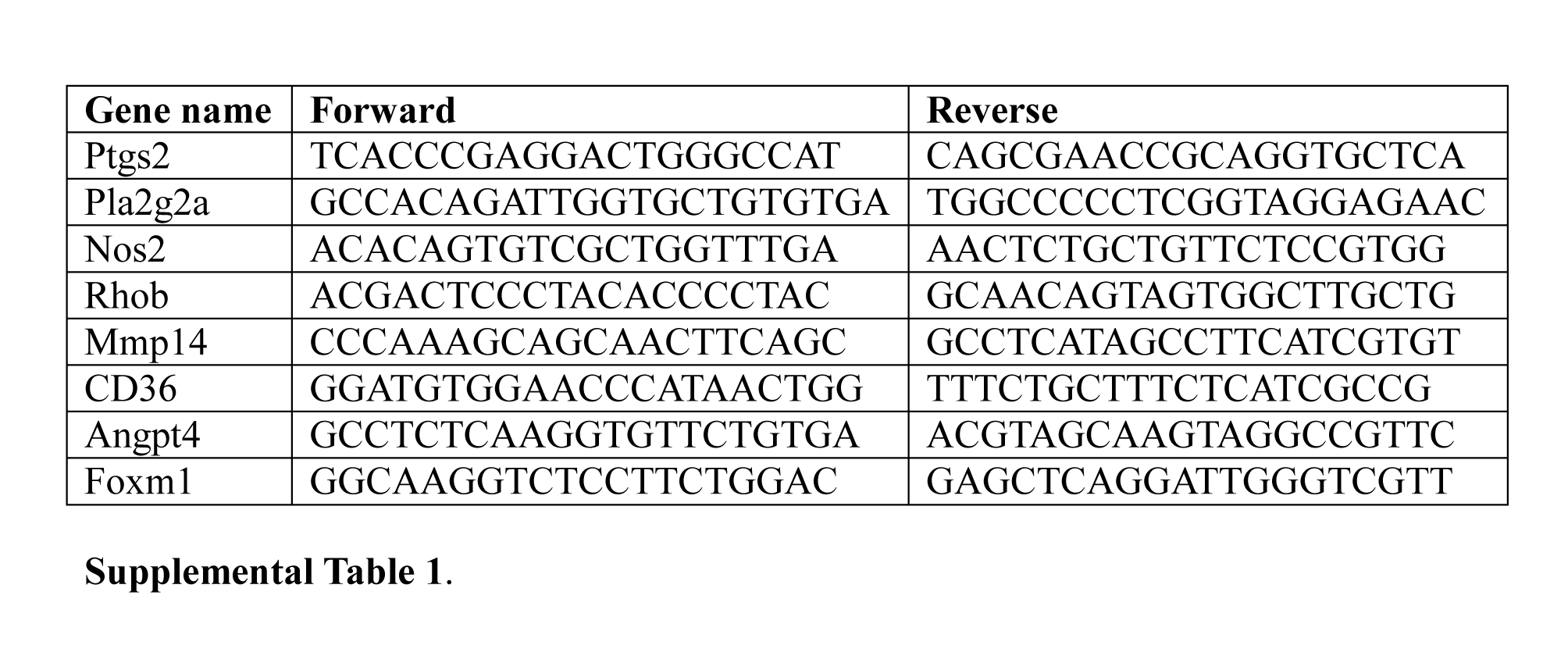

Supplement: Table S1 — List of Primer oligonucleotide sequences used for qPCR experiments. (TIF) [file pone.0083762.s006.tif]
